# Communication Efficient Federated Generalized Tensor Factorization for Collaborative Health Data Analytics

**DOI:** 10.1145/3442381.3449832

**Published:** 2021-04

**Authors:** Jing Ma, Qiuchen Zhang, Jian Lou, Li Xiong, Joyce C. Ho

**Affiliations:** Emory University

**Keywords:** Electronic Health Records (EHR), Tensor Factorization, Federated Learning, Computational Phenotyping

## Abstract

Modern healthcare systems knitted by a web of entities (e.g., hospitals, clinics, pharmacy companies) are collecting a huge volume of healthcare data from a large number of individuals with various medical procedures, medications, diagnosis, and lab tests. To extract meaningful medical concepts (i.e., phenotypes) from such higher-arity relational healthcare data, tensor factorization has been proven to be an effective approach and received increasing research attention, due to their intrinsic capability to represent the high-dimensional data. Recently, federated learning offers a privacy-preserving paradigm for collaborative learning among different entities, which seemingly provides an ideal potential to further enhance the tensor factorization-based collaborative phenotyping to handle sensitive personal health data. However, existing attempts to federated tensor factorization come with various limitations, including restrictions to the classic tensor factorization, high communication cost and reduced accuracy. We propose a *communication efficient* federated *generalized* tensor factorization, which is flexible enough to choose from a variate of losses to best suit different types of data in practice. We design a three-level communication reduction strategy tailored to the generalized tensor factorization, which is able to reduce the uplink communication cost up to 99.90%. In addition, we theoretically prove that our algorithm does not compromise convergence speed despite the aggressive communication compression. Extensive experiments on two real-world electronics health record datasets demonstrate the efficiency improvements in terms of computation and communication cost.

## 1 INTRODUCTION

Recent years have witnessed an unprecedented growth of health data (e.g., in the form of EHR, electronic health records) being collected from a variety of institutions, including hospitals, clinics, pharmaceutical companies, and health insurance providers. Computational phenotyping, the process of extracting meaningful and concise medical concepts (i.e., phenotypes) from the health data, is an indispensable stepping stone towards in-depth medical decisionmaking, including precision medicine, influenza surveillance, drug discovery, to name a few. Computational phenotyping is known to be challenging, given the fact that health data are collected from a large number of individuals with each one’s medical record consisting of various of medical procedures, medications, diagnosis and lab tests. That is, the health data is massive and multidimensional. In addition, in order to collaboratively learn phenotypes from the data belonging to different institutes (known as collaborative phenotyping), the sensitive nature of the health data serves as an additional restriction.

To learn phenotypes from the multidimensional EHR data, tensor factorization has received increasing interest [12–14, 20, 27, 28, 36]. Tensor has the intrinsic capability to succinctly represent the multidimensional data [21] and has applications beyond health data analytics, e.g., recommender systems [18], spatio-temporal data analysis [26], computer vision [35], and signal processing [32]. The CANDECOMP/PARAFAC or canonical polyadic (CP) tensor factorization (TF) [7, 11] and its generalization GTF [15] are fundamental tools for analyzing the tensors. Despite their effectiveness and wide applications, the scalability is often a major issue preventing it from being applied to larger scale health datasets, which are commonly encountered nowadays. To improve the scalability of TF, distributed tensor factorization (DTF) methods [6, 9, 12, 20, 27, 31, 41] are capable of processing large tensors that cannot be dealt by a single machine. It also complies with the practical scenario for the health data which is collected and held across multiple physically distributed medical institutions.

Most recently, federated tensor factorization (FTF) methods [20, 27] are proposed as a better DTF paradigm for decentralized data in terms of privacy protection, while maintaining similar computational and storage scalability. It avoids communicating both the raw tensor and individual mode related variables to the server, which shares the same spirit of the more general federated learning [17], i.e., to learn a joint model across all the clients without communicating individual-level data. By avoiding sharing the raw tensor and the patient mode related variables (e.g., patient factor and partial gradient along the patient mode), FTF offers better patient privacy protection.

Besides computational complexity and alleviating storage usage which are the focus of most existing DTF methods, the communication overhead can be a third important bottleneck, especially for the federated setting, where the participating institutions do not have a dedicated communication network for communication purposes, e.g., hospitals, clinics. Considering the asymmetric bandwidths, the uplink communication (i.e. the communication from the client to the server) can quickly become the bottleneck preventing these clients from participating in the FTF. In federated computational phenotyping, due to the great variety of the attributes (e.g., types of medication can be thousands), the high dimensional tensor incurs high communications cost to communicate the intermediate variables during each communication cycle.

### 1.1 Contributions

In this paper, we investigate how to reduce the uplink communication cost of the federated tensor factorization-based collaborative phenotyping with guaranteed convergence and quality preservation. It is a challenging task, especially considering the communication efficiency issue is under studied in the broader distributed tensor factorization literature. To be more flexible and suitable for a variety of applications, we consider the federated generalized tensor factorization (FGTF), which greatly extends the existing federated classic TF [20, 27].

First, we aim to reduce the uplink communication cost in each communication round. We design a two-level per-round communication reduction strategy: block-level and element-level, which reduce $\left(1-\frac{1}{D}\right)$ and over 96.8% of the uplink communication, correspondingly, where *D* is the number of blocks. For the block-level, we exploit the multi-factor structure of TF/GTF by utilizing the randomized block update. It enables each client to send only the partial gradient of the sampled block, rather than the full gradient of all blocks. For the element-level, we introduce gradient compression techniques, which have found success in deep learning training [2, 4, 19, 37, 42], to compress each element of the communicated partial gradient from the floating point representation to low-precision representation. Since there exists error between the true partial gradient and the compressed one, the convergence can be slower and the output quality can be lower. We further introduce the error-feedback mechanism [19] which records such error and feeds it back to restore the shift.

With both levels of per-round communication reduction, we propose the federated GTF with communication compression and error-feedback (**FedGTF-EF**). We analyze the convergence of **FedGTF-EF** and obtain the $O\left(\frac{1}{\sqrt{T}}\right)$ rate after *T* iterations (Thm. 4.1) under common and mild assumptions (Assumptions 4.1–4.5). The convergence is equivalent to the distributed stochastic gradient descent (SGD) with full precision gradient communication and distributed SGD with gradient compression and error-feedback [42]. In addition, since constraints and nonsmooth regularizations are common in GTF, we further extend the convergence result to the proximal setting (4.2) where the additional “simple regularizer” in Assumption 4.6 is satisfied. Compared to the existing analysis with gradient compression and error-feedback, our convergence analysis accounts for both the block randomized update strategy and the proximal operation.

Second, we reduce the number of communication rounds to further reduce the uplink communication. To do so, we introduce periodic communication [4, 23, 33] into **FedGTF-EF** and denote this algorithm as **FedGTF-EF-PC**, in which the clients send the update to the server after *τ* > 1 local iterations instead of communicating after every iteration. A key question is whether the periodic communication will slow down the convergence. If so, the number of iterations will increase and the overall number of communications may not reduce. We analyze the convergence of **FedGTF-EF-PC** in Thm. 4.3 and obtain the same convergence $O\left(\frac{1}{\sqrt{T}}\right)$ rate with **FedGTF-EF** under the same set of assumptions. This indicates that **FedGTF-EF-PC** can indeed further reduce the uplink communication cost by $1-\frac{1}{\tau }$. As a result, our proposed **FedGTF-EF-PC** can reduce up to $1-\frac{1}{32D\tau }$ uplink communication cost if the Sign compressor (Def.2.1) is used.

Third, we evaluate **FedGTF-EF** and **FedGTF-EF-PC** in the federated collaborative phenotyping task. We conduct experiments on two real-world EHR datasets, which show that the proposed method can effectively reduce uplink communication cost (99.90% reduction), without compromising convergence and factorization quality.

## 2 PRELIMINARIES AND BACKGROUND

### 2.1 Notation

The frequently used notation in this paper is summarized in Table 1. We denote an order *D* tensor by $X\in {\mathbb{R}}^{I_{1}\times \ldots \times I_{D}}$, its ($i_{1},\ldots ,i_{D}$)-th element by MATLAB representation $\text{X}\left(i_{1},\ldots ,i_{D}\right)$. Let I denote the index set of all tensor entries, $|I|=I_{\text{Π}}=\prod_{d=1}^{D}I_{d}$. The mode-*d* unfolding (also called matricization) is denoted by ${\text{X}}_{<d>}\in {\mathbb{R}}^{I_{d}\times I_{\text{Π}}/I_{d}}$, where $\left({\text{X}}_{<d>}\right)\left(i_{d},j\right)$ and $X\left(i_{1},i_{2},\ldots ,i_{D}\right)$ has the **index mapping:** $j=1+\sum_{\begin{array}{l} k=1,\\ k\neq d \end{array}}^{D}\left(i_{k}-1\right)J_{k}$, $J_{k}=\prod_{\begin{array}{c} q=1.\\ q\neq d \end{array}}^{k-1}I_{q}$. Each column ${\text{X}}_{<d>}(:,j)$ is called a mode-*d* fiber of X.

### 2.2 Generalized Tensor Factorization

**Figure 1:**
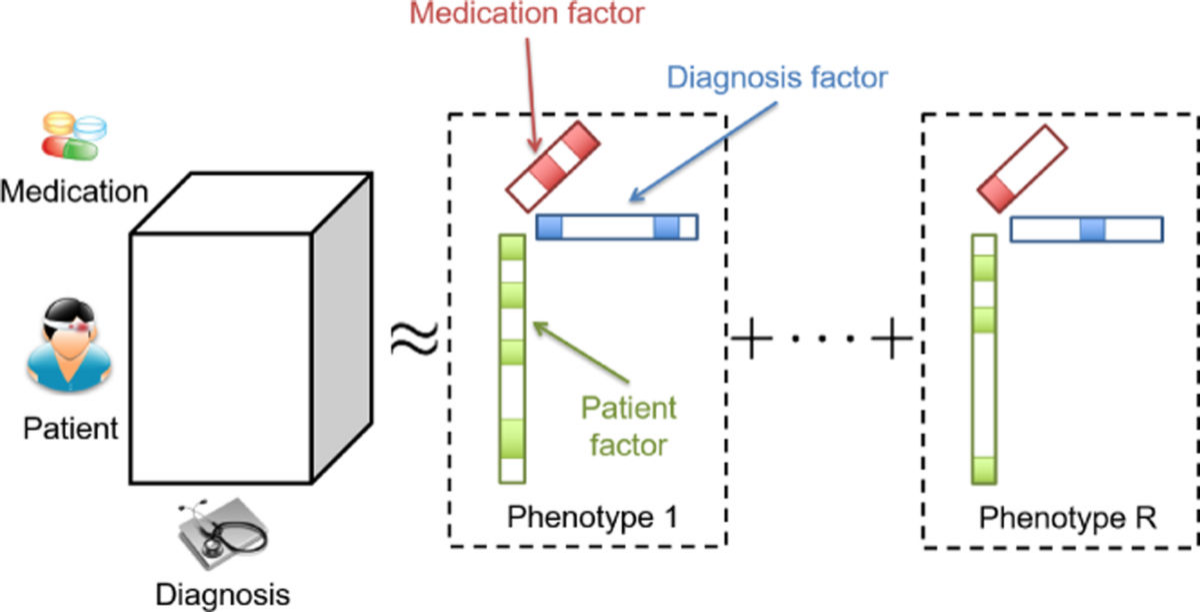
Illustration of EHR tensor and phenotype extraction via tensor factorization [14].

As illustrated in Figure 1, let us consider the EHR tensor $X\in {\mathbb{R}}^{I_{1}\times ,\ldots ,\times I_{D}}$, which consists of patient mode (*I*_1_), diagnosis mode (*I*_2_), medication mode (*I*_3_), and so on. The regularized Generalized CANDECOMP-PARAFAC (GTF) [15] extracts the phenotypes by decomposing the EHR tensor into *R* phenotyps, where each consists of a patient factor, diagnosis factor, and a medication factor. GTF has the following objective function:

(1) $$\begin{array}{c} \arg\underset{A}{\min}F(A,X)=\sum_{i\in I}f(A(i),X(i))+\sum_{d=1}^{D}r_{d}\left({\text{A}}_{\left(d\right)}\right),\\ \text{s.t.}\;A=\sum_{i=1}^{R}{\text{A}}_{\left(1\right)}(:,i)\circ \ldots \circ {\text{A}}_{\left(D\right)}(:,i), \end{array}$$

which breaks down into three parts:

1. Factorization constraint: The constraint of $A=\sum_{i=1}^{R}{\text{A}}_{\left(1\right)}(:,i)\circ \ldots \circ {\text{A}}_{\left(D\right)}(:,i)$ approximates the low-rank CP tensor $A\in {\mathbb{R}}^{I_{1}\times ,\ldots ,\times I_{D}}$ as the sum of *R* rank-one tensors, where ${\text{A}}_{\left(d\right)}\in {\mathbb{R}}^{I_{d}\times R}$ is the *d*-th factor matrix and ${\text{A}}_{\left(d\right)}(:,i)$ is its *i*-th column. For phenotyping, ${\text{A}}_{\left(1\right)},{\text{A}}_{\left(2\right)},{\text{A}}_{\left(3\right)}$ correspond to the patient factor, diagnosis factor, and medication factor, correspondingly.
2. Element-wise loss function: f(A(i),X(i)) is the element-wise loss between the low-rank CP tensor A and the input tensor X. For the classic CP [7, 11], $f(A(i),X(i)):=\frac{1}{2}{\left(A(i)-X(i)\right)}^{2}$, which is the least square loss. GCP is more generalized in the sense that the loss function can take other forms to best suit the property of the input tensor. For example, f(⋅) can be chosen based on the distribution of the tensor entries, e.g. logit loss for binary data: $f_{\text{logit}}=\log(1+A(i))-X(i)A(i)$, for all i∈I, or f(⋅) can be the Huber loss for robustness purpose.
3. Regularization: $r_{d}(\cdot )$ is the regularization applied to the factor ${\text{A}}_{d}$, which can be the smooth ${\left\|{\text{A}}_{\left(d\right)}\right\|}_{F}^{2}$ norm or the nonsmooth ${\left\|{\text{A}}_{\left(d\right)}\right\|}_{1}$ norm. In practice, the regularization can improve the interpretability of the phenotypes.

#### Existing federated computational phenotyping.

Two recent papers [20] and [27] consider federated tensor factorization and apply it to the federated phenotyping. They have the following limitations. 1) Both are limited to the CP model and [20] applies least square solver as its client side local updater, which is difficult to be extended to more general losses other than least square loss. 2) Although extensible to using infrequent communication, each communication round still incurs high communication cost since both requires sending all factors in full precision. In addition, [20] also requires communication of the Lagrangian dual variables which doubles the communication cost. 3) Both alter the original objective function by introducing extra terms to enforcing consensus of factors among all clients: [20] introduces linear constraint and transforms it to Lagrangian dual formulation while [27] introduces elastic penalty terms. These terms can lead the extracted factors to deviate from the centralized solution, thus negatively impacting the phenotyping accuracy.

### 2.3 SGD with Gradient Compression, Error-Feedback and Periodic Communication

#### Gradient Compression.

Recently, one of the most successful approaches to mitigating the communication overhead is via gradient compression, which compresses the gradient to be communicated from the full precision representation (e.g. float or double number representation) to a much lower precision representation (e.g. aggressively compressed to 1-bit). The following definition introduces one of the most popular compressors:

#### Definition 2.1.

(Sign Compressor) For an input tensor $\text{x}\in {\mathbb{R}}^{d}$, its compression via Sign(⋅) is $\mathrm{Sign}(\text{x})=∥\text{x}{∥}_{1}/d\cdot sign(\text{x})$, where sign takes the sign of each element of x.

#### Error-Feedback.

Due to aggressive compression, the algorithm can converge slower (or even diverge) compared to the full precision counterpart. The main cause is the error between the full precision gradient and the compressed one. Error-feedback [19, 34, 42] is a technique that memorizes this error in the current iteration and feeds it back to the gradient of the next iteration. By doing so, it can rigorously guarantee uncompromised convergence compared to the full-precision SGD.

#### Periodic Communication.

Instead of reducing the communication cost per-communication round, periodic communication or local SGD [23, 33] reduces it by decreasing the communication frequency in hope that the total number of communications rounds can be reduced. Each clients will execute *τ* > 1 local updates before communicating to the server. [4] shows that it is possible to combine communication compression and periodic communication together. [34] provides a unified framework by error-feedback to analyze the convergence of gradient compression and local SGD.

## 3 PROPOSED METHODS

**Figure 2:**
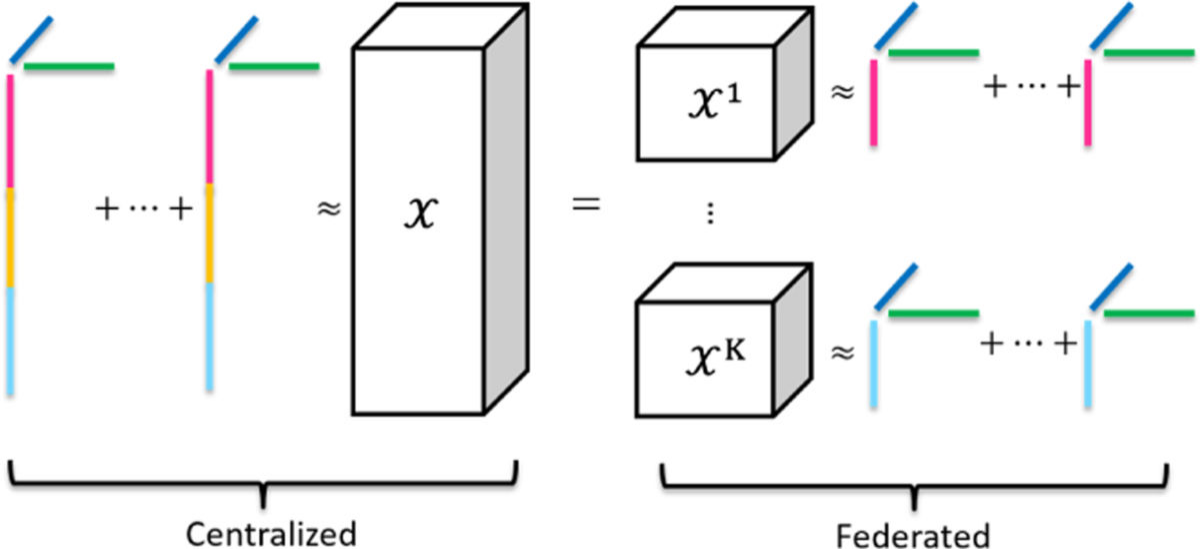
Illustration of collaborative phenotyping via federated tensor factorization [20].

Under the federated setting as illustrated in Fig. 2, the EHR tensor $X\in {\mathbb{R}}^{I_{1}\times ,\ldots ,\times I_{D}}$ will be collectively held by *K* institutions. The *k*-th client’s local tensor is denoted by $X^{k}\in {\mathbb{R}}^{I_{1k}\times I_{2}\times \ldots \times I_{D}}$, which contains information about $I_{1^{k}}$ individuals, such that $\sum_{k=1}^{K}I_{1^{k}}=I_{1}$. That is, we consider the horizontally partitioned setting where different hospitals share the same feature space. We also note that there are related works addressing other settings like vertically partitioned settings [8, 24, 25, 39] which are complementary to our work. The aim of the federated computational phenotying is to collaboratively compute the phenotyes from EHR tensor across *K* institutions without sharing the raw tensor and patient mode variables. The objective function of the federated GTF is as follows

(2) $$\begin{array}{c} \underset{\left({\text{A}}_{\left(1\right)},\ldots ,{\text{A}}_{\left(D\right)}\right)}{\text{argmin}}\sum_{k=1}^{K}F\left(A,X^{k}\right)+\sum_{d=1}^{D}r_{d}\left({\text{A}}_{\left(d\right)}\right),\\ \text{s.t.}\;A={\text{A}}_{\left(1\right)}\circ \ldots \circ {\text{A}}_{\left(D\right)}. \end{array}$$

**Figure 3:**
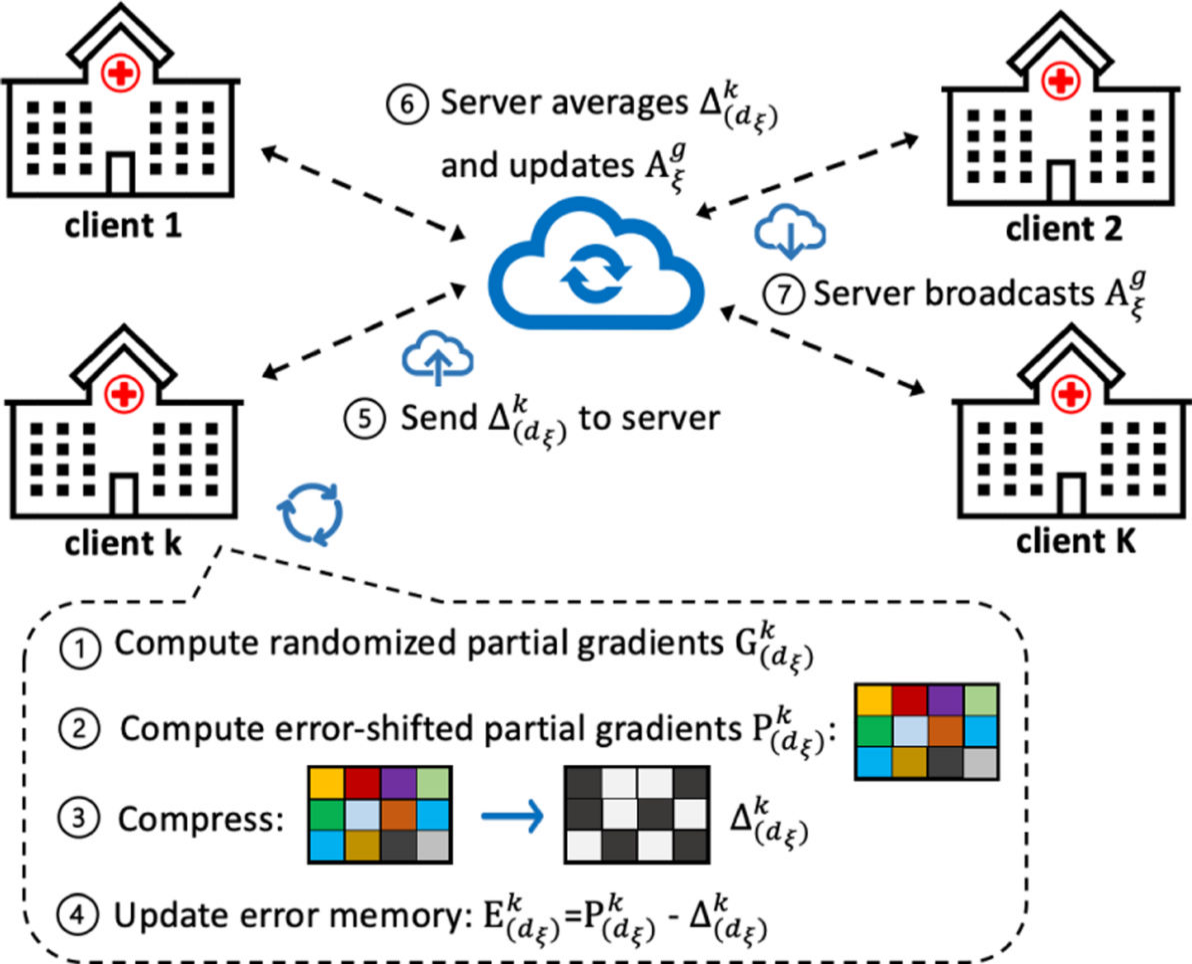
Illustration of the execution of FedGTF-EF and FedGTF-EF-PC.

In fact, the above formulation can be extended to general multi-block problems as well. Thus, our algorithms are not limited to federated GTF problems but also to other nonconvex problems possessing a multi-block decision variable structure, e.g. [40]. In the following, we propose the federated generalized tensor factorization with communication efficiency improvements via block randomization, gradient compression, error feedback and periodic communication. The execution of the proposed algorithm is illustrated in Fig. 3.

### 3.1 FedGTF-EF: Communication Efficient GTF with Block Randomization, Gradient Compression and Error-Feedback

We reduce the uplink communication in each communication round at two levels: block-level and element-level. The detailed algorithm is displayed in Algorithm 1 with functionalities of key steps annotated. At the block-level, to avoid sending all factors, we use a randomized block (i.e., randomized factor) update, which only requires the communication of the partial gradient of the factor being sampled (the computation of the partial gradient will be detailed in Sec.3.3). At the element-level, we compress each element of the communication to a low-precision representation before sending to the server (Line 6). Each client *k* keeps *D* local pairs of ${\text{P}}_{\left(d\right)}^{k}$ (the error-shifted full-precision partial gradient), ${\text{Δ}}_{\left(d\right)}^{k}$ (the compressed gradient to be communicated), ${\text{E}}_{\left(d\right)}^{k}$ (error record between the full precision gradient and the compressed gradient), for all *d* = 1,...,*D* factors. Depending on whether the regularizer is smooth or not, either simple gradient descent (Line 8) or proximal gradient descent (Line 9) can be chosen to update the sampled factor, respectively.

**Algorithm 1:** FedGTF-EF: Communication Efficient GTF with Block Randomization, Gradient Compression and Error-Feedback

**Input:**X,γ[t],A[0], randomized block sampling sequence $d_{\xi }[0],\ldots ,d_{\xi }[T]$;	
1:	**for** t=0,…,T **do**
2:	**On Each Client Nodes** k∈1,…,K:
3:	**if** $d=d_{\left(\xi \right)}[t]$ **then**
4:	Compute stochastic gradient ${\text{G}}_{\left(d\right)}^{k}[t]$ by eq.(4);
5:	${\text{P}}_{\left(d\right)}^{k}[t]=\gamma [t]{\text{G}}_{\left(d\right)}^{k}[t]+{\text{E}}_{\left(d\right)}^{k}[t]$; %% error feedback
6:	${\text{Δ}}_{\left(d\right)}^{k}[t]=\text{Compress}\;\left({\text{P}}_{\left(d\right)}^{k}[t]\right)$, Send ${\text{Δ}}_{\left(d\right)}^{k}[t]\left(\text{i.e.}\;{\text{Δ}}_{\left(d_{\xi }[t]\right)}^{k}[t]\right)$ to the server; %% compression
7:	Receive $\frac{1}{K}\sum_{k=1}^{K}{\text{Δ}}_{\left(d\right)}^{k}[t]$ (i.e. $\frac{1}{K}\sum_{k=1}^{K}{\text{Δ}}_{\left(d_{\xi }[t]\right)}^{k}[t]$) from the server;
8:	Smooth regularization case: ${\text{A}}_{\left(d\right)}[t+1]={\text{A}}_{\left(d\right)}[t]-\frac{1}{K}\sum_{k=1}^{K}{\text{Δ}}_{\left(d\right)}^{k}[t]$; %% update factor
9:	Nonsmooth regularization case: ${\text{A}}_{\left(d\right)}[t+1]={\text{Prox}}_{r_{d}}\left({\text{A}}_{\left(d\right)}[t]-\frac{1}{K}\sum_{k=1}^{K}{\text{Δ}}_{\left(d\right)}^{k}[t]\right)$;
10:	${\text{E}}_{\left(d\right)}^{k}[t+1]={\text{P}}_{\left(d\right)}^{k}[t]-{\text{Δ}}_{\left(d\right)}^{k}[t]$; %% update error memory
11:	**else if** $d\neq d_{\xi }[t]$ **then**
12:	${\text{A}}_{\left(d\right)}[t+1]={\text{A}}_{\left(d\right)}[t],{\text{E}}_{\left(d\right)}^{k}[t+1]={\text{E}}_{\left(d\right)}^{k}[t]$; %% unselected blocks are kept unchanged
13:	**end if**
14:	**On Server Node:**
15:	Receive ${\text{Δ}}_{\left(d_{\xi }[t]\right)}^{k}[t]$ from all client nodes; Broadcast $\frac{1}{K}\sum_{k=1}^{K}{\text{Δ}}_{\left(d_{\xi }[t]\right)}^{k}[t]$ to all client nodes;
16:	**end for**

### 3.2 FedGTF-EF-PC: Further Communication Reduction by Periodic Communication

We further reduce the uplink communication cost by introducing a third communication compression level: round level. That is, we decrease the communication frequency from one iteration per-communication to *τ* > 1 iterations per-communication, which manifests a periodic communication behaviour [4, 23, 33]. The detailed algorithm is provided in Algorithm 2. The major difference with Algorithm 1 is that each client compresses and sends the collective updates across *τ* iterations (Line 9–10), instead of the partial gradient in a single iteration. The error feedback (Line 9) and error memory (Line 7, 13) are adjusted accordingly.

### 3.3 Efficient Partial Stochastic Gradient Computation for FedGTF

After presenting the overall algorithms, we now present an efficient partial stochastic gradient computation subroutine to compute ${\text{G}}_{\left(d\right)}^{k}[t]$ in Step 1 of Fig. 3 and Line 4 of Algorithm 1 and 2. The first mode (i.e., *I*_1_) is the individual mode (e.g., patient mode) which can be kept local to each client. Thus, when $d_{\xi }[t]=1$, we skip the communication, which not only further reduces the communication cost, but also is beneficial to the privacy since the individual-level information is not shared.

Next, we specify the computation of the partial stochastic gradient ${\text{G}}_{\left(d\right)}^{k}[t]$ based on the efficient fiber sampling technique [5, 10]. The deterministic partial gradient is $\nabla_{{\text{A}}_{\left(d\right)}}F(\text{A})={\text{Y}}_{<d>}{\text{H}}_{d}$ [15], where ${\text{H}}_{d}\in {\mathbb{R}}^{I_{\text{Π}}/I_{d}\times R}$ is the mode-*d* Khatri-Rao product of the all

**Algorithm 2:** FedGTF-EF-PC: Further Reducing Communication Cost by Periodic Communication

**Input:**$X,\gamma [t],\text{A}[0],{\text{A}}^{k}[0]=\text{A}[0],\forall k=1,\ldots ,K$, randomized block sampling sequence $d_{\xi }[0],\ldots ,d_{\xi }[T]$;	
1:	**for**t=0,…,T**do**
2:	**On Each Client Nodes** k∈1,…,K:
3:	**if** $d=d_{\left(\xi \right)}[t]$ **then**
4:	Compute stochastic gradient ${\text{G}}_{\left(d\right)}^{k}[t]$ by eq.(4);
5:	${\text{A}}_{\left(d\right)}^{k}\left[t+\frac{1}{2}\right]={\text{A}}_{\left(d\right)}^{k}[t]-\gamma [t]{\text{G}}_{\left(d\right)}^{k}[t]$; %% local update by stochastic gradient descent
6:	**if** (tmodτ)≠0 **then**
7:	${\text{E}}_{\left(d\right)}^{k}[t+1]={\text{E}}_{\left(d\right)}^{k}[t],{\text{A}}_{\left(d\right)}^{k}[t+1]={\text{A}}_{\left(d\right)}^{k}\left[t+\frac{1}{2}\right],{\text{A}}_{\left(d\right)}^{g}[t+1]={\text{A}}_{\left(d\right)}^{g}[t]$; %% no communication
8:	**else**
9:	${\text{P}}_{\left(d\right)}^{k}[t]=\left({\text{A}}_{\left(d\right)}^{g}[t]-{\text{A}}_{\left(d\right)}^{k}\left[t+\frac{1}{2}\right]\right)+{\text{E}}_{\left(d\right)}^{k}[t]$; %% error feedback to accumulated update
10:	${\text{Δ}}_{\left(d\right)}^{k}[t]=\text{Compress}\left({\text{P}}_{\left(d\right)}^{k}[t]\right)$, Send ${\text{Δ}}_{\left(d\right)}^{k}[t]\;\text{(i.e.}\;{\text{Δ}}_{\left(d_{\xi }[t]\right)}^{k}[t])$ to the server;
11:	Receive ${\text{A}}_{\left(d\right)}^{g}[t+1]$ from the server, ${\text{A}}_{\left(d\right)}^{k}[t+1]={\text{A}}_{\left(d\right)}^{g}[t+1]$; %% compression
12:	**end if**
13:	${\text{E}}_{\left(d\right)}^{k}[t+1]={\text{P}}_{\left(d\right)}^{k}[t]-{\text{Δ}}_{\left(d\right)}^{k}[t]$; %% update error memory
14:	**else if** $d\neq d_{\xi }[t]$ **then**
15:	${\text{A}}_{\left(d\right)}^{k}[t+1]={\text{A}}_{\left(d\right)}^{k}[t],{\text{E}}_{\left(d\right)}^{k}[t+1]={\text{E}}_{\left(d\right)}^{k}[t]$;
16:	**end if**
17:	**On Server Node:**
18:	Receive ${\text{Δ}}_{\left(d_{\xi }[t]\right)}^{k}[t]$ from all client nodes; Broadcast ${\text{A}}_{\left(d_{\xi }[t]\right)}^{g}[t+1]={\text{A}}_{\left(d_{\xi }[t]\right)}^{g}[t]-\frac{1}{K}\sum_{k=1}^{K}{\text{Δ}}_{\left(d_{\xi }[t]\right)}^{k}[t]$ to all client nodes;
19:	**end for**

factors except the *d*-th, i.e. ${\text{H}}_{d}={\text{A}}_{\left(D\right)}⊙\ldots ⊙{\text{A}}_{\left(d+1\right)}⊙{\text{A}}_{\left(d-1\right)}\cdots ⊙{\text{A}}_{\left(1\right)}$; and ${\text{Y}}_{<d>}$ is the *d*-unfolding of the element-wise partial gradient $Y\in {\mathbb{R}}^{I_{1}\times \ldots \times I_{D}}$, where $Y(i)=\frac{\partial f(A(i),X(i))}{\partial A(i)}$, for all i∈I. We approximate $\nabla_{{\text{A}}_{\left(d\right)}}F(\text{A})$ by sampling |S| fibers (i.e. |S| columns of ${\text{Y}}_{\left(d\right)}$) and the corresponding |S| rows of ${\text{H}}_{d}$, where S denotes the index of the sampled fibers. The stochastic partial gradient is then

(3) $${\text{G}}_{\left(d\right)}[t]={\text{Y}}_{<d>}(:,S){\text{H}}_{d}(S,:),$$

where both ${\text{Y}}_{<d>}(:,S)$ and ${\text{H}}_{d}(S,:)$ can be efficiently computed, because: 1) the computation of ${\text{Y}}_{<d>}(:,S)$ only involves $I_{d}\times |S|$ element-wise partial gradient computation [22] and 2) the computation of ${\text{H}}_{d}(S,:)$ can be obtained without forming the full Khatri-Rao product of ${\text{H}}_{d}$ [32]. For the *s*-th row of ${\text{H}}_{d}$, its index $\left(i_{1}^{s},\ldots ,i_{D}^{S}\right)$ can be obtained by the **index mapping** in Section 2.1. Then, $\text{H}(s,:)={\text{A}}_{\left(1\right)}\left(i_{1}^{s},:\right)⊛\ldots ⊛{\text{A}}_{\left(d-1\right)}\left(i_{d-1}^{s},:\right)⊛{\text{A}}_{\left(d+1\right)}\left(i_{d+1}^{s},:\right)⊛\ldots ⊛{\text{A}}_{\left(D\right)}\left(i_{D}^{S},:\right)$, where ⊛ is the Hadamard product. Finally, the local stochastic gradient ${\text{G}}_{\left(d\right)}^{k}[t]$ can be efficiently computed by substituting its local tensor partition Y^*k*^ and local factors ${\text{A}}_{\left(d\right)}^{k}$ into eq.(3), which gives

(4) $${\text{G}}_{\left(d\right)}^{k}[t]={\text{Y}}_{<d>}^{k}(:,S){\text{H}}_{d}^{k}(S,:),$$

where ${\text{H}}^{k}(s,:)={\text{A}}_{\left(1\right)}^{k}\left(i_{1}^{s},:\right)⊛\ldots ⊛{\text{A}}_{\left(d-1\right)}^{k}\left(i_{d-1}^{s},:\right)⊛{\text{A}}_{\left(d+1\right)}^{k}\left(i_{d+1}^{s},:\right)⊛\ldots ⊛A_{\left(D\right)}^{k}\left(i_{D}^{s},:\right)$. According to the complexity analysis, our gradient computation in eq.(4) matches the state-of-the-art efficiency of GTF computation, e.g., [10].

## 4 ALGORITHM ANALYSIS

This section presents the convergence analysis and complexity analysis of FedGTF-EF and FedGTF-EF-PC. A proof sketch of the convergence analysis is provided in the appendix.

### 4.1 Convergence Analysis

#### 

##### Assumptions.

In order to analyze the convergence, we make the following assumptions which are common to many machine learning problems [4, 10, 34, 42]. Let the randomness of computing stochastic gradient of ${\text{G}}_{\left(d_{\xi }[t]\right)}[t]$ be ζ[t], the randomness of sampling the block be ξ[t], the filtration upon iteration *t* be F[t]={ζ[0],ξ[0],…,ζ[t−1],ξ[t−1]}.

###### Assumption 4.1.

*(Block-wise Smoothness of the Loss Function)*F(⋅)*is L*_(*d*)_-*block-wise smooth, ford* = 1,...,*D, i.e. for all* $\text{A},\text{B},F(\text{B})\leq F(\text{A})+\langle \nabla_{{\text{A}}_{\left(d\right)}},{\text{B}}_{\left(d\right)}-{\text{A}}_{\left(d\right)}\rangle +\frac{L_{\left(d\right)}}{2}{\left\|{\text{B}}_{\left(d\right)}-{\text{A}}_{\left(d\right)}\right\|}_{F}^{2}$.

###### Assumption 4.2.

*(Unbiased Gradient Estimation) The stochastic gradient is unbiased:*$E_{\zeta [t]}\left[{\text{G}}_{d_{\xi [t]}}^{k}[t]|F[t],\xi [t]\right]=\nabla_{{\text{A}}_{\left(d_{\xi [t]}\right)}}F(\text{A}[t])$.

###### Assumption 4.3.

*(Bounded Variance) The stochastic gradient has bounded variance:*

$$E_{\zeta [t]}\left[{\left\|{\text{G}}_{\left(d_{\xi }[t]\right)}^{k}[t]-\nabla_{{\text{A}}_{\left(d_{\xi }[t]\right)}}F(\text{A}[t])\right\|}_{F}^{2}|F[t],\xi [t]\right]\leq \sigma_{d}^{2}.$$

###### Assumption 4.4.

*(Bounded Gradient)*${\left\|\nabla_{{\text{A}}_{\left(d\right)}}F(\text{A}[t])\right\|}_{F}^{2}\leq \omega_{d}^{2}$.

###### Assumption 4.5.

*(δ-approximated Compression [19]) An operator* Compress : ${\mathbb{R}}^{d}\to {\mathbb{R}}^{d}$ *is an δ-approximate compressor for δ* ∈ (0, 1] *if* ${\left\|\text{Compress}(\text{x})-\text{x}\right\|}_{2}^{2}\leq (1-\delta ){\left\|\text{x}\right\|}_{2}^{2}$.

Many compressors satisfy the above condition [4]: top-k or random k-sparsification, stochastic k-level quantization, stochastic rotated quantization and the Sign compressor in Definition 2.3.

###### Assumption 4.6.

*(Simple Regularization Function) The regularization functions*$r_{d}(\cdot ),d=1,\ldots ,D$, *are convex, lower semi-continuous and admit closed-form proximal operator:*

$${\text{Prox}}_{r_{d}}\left({\text{B}}_{d}\right)={\text{argmin}}_{{\text{A}}_{\left(d\right)}}\frac{1}{2}{\left\|{\text{A}}_{\left(d\right)}-{\text{B}}_{\left(d\right)}\right\|}_{F}^{2}+r_{d}\left({\text{A}}_{\left(d\right)}\right).$$

Many common regularizations satisfy this assumption, for example, the *ℓ*_1_-norm for inducing sparsity which has the soft-thresholding operator as its proximal operator.

#### 4.1.1 Convergence Analysis of Algorithm 1.

##### Smooth regularization case.

To prove the convergence, we extend the delayed gradient perspective in [19] to our block randomized setting by introducing the following virtual variables only for the proof: ${\tilde{\text{A}}}_{\left(d\right)}[t]:={\text{A}}_{\left(d\right)}[t]-\frac{1}{K}\sum_{k=1}^{K}{\text{E}}_{\left(d\right)}^{k}[t]$. Then, we have the following virtual recurrence: if $d=d_{\xi }[t],{\tilde{\text{A}}}_{\left(d\right)}[t+1]={\text{A}}_{\left(d\right)}[t+1]-\frac{1}{K}\sum_{k=1}^{K}{\text{E}}_{\left(d\right)}[t+1]={\tilde{\text{A}}}_{\left(d\right)}[t]-\gamma [t]\frac{1}{K}\sum_{k=1}^{K}{\text{G}}_{\left(d\right)}^{k}[t]$; else if $d\neq d_{\xi [t]},{\tilde{\text{A}}}_{\left(d\right)}[t+1]={\tilde{\text{A}}}_{\left(d\right)}[t]$. Thus, the recurrence can be viewed as the block randomized SGD with the variable ${\tilde{\text{A}}}_{\left(d\right)}[t]$ which corresponds to ${\text{A}}_{\left(d\right)}[t]$ with delayed information $\frac{1}{K}\sum_{k=1}^{K}{\text{E}}_{\left(d\right)}^{k}[t]$ added. The convergence of Algorithm 1 applied to the smooth smooth regularization is as follows.

###### Theorem 4.1.

*Suppose that Assumptions 4.1-4.5 hold. Let*$\left({\text{A}}_{\left(1\right)}[t],\ldots ,{\text{A}}_{\left(D\right)}[t]\right)$*be the iterates of Algorithm 1 with Line 8. Let*$\gamma =\min\left\{\frac{1}{2L},\frac{\varrho }{\sqrt{T+1}/\sqrt{K}+\frac{{\left(1-\delta \right)}^{1/3}}{\delta^{2/3}}T^{1/3}}\right\}$, *for some*ϱ>0. *We have*

$$\begin{array}{l} E\left[\frac{1}{D}\sum_{d=1}^{D}∥\nabla_{{\text{A}}_{\left(d\right)}}F(\text{A}[\text{Output}]){∥}_{F}^{2}\right]\\ \leq \frac{8L}{T+1}\left(F(\text{A}[0])-F^{*}\right)+\left[\frac{4}{\varrho }\left(F(\text{A}[0])-F^{*}\right)+\frac{2L\sigma^{2}\varrho }{D}\right]\frac{1}{\sqrt{M(T+1)}}\\ +\left[\frac{4}{\varrho }\left(F(\text{A}[0])-F^{*}\right)+\frac{8L^{2}\varrho^{2}\left(\sigma^{2}+\omega^{2}\right)}{D}\right]\frac{{\left(1-\delta \right)}^{1/3}}{\delta^{2/3}{\left(T+1\right)}^{2/3}}, \end{array}$$

*where*$\text{A[Output}]=\left({\text{A}}_{\left(1\right)}[\text{Output}],\ldots ,{\text{A}}_{\left(D\right)}[\text{Output}]\right)$*is sampled from*A[0]*to*A[T]*with uniform distribution*, $F^{*}$*is the optimal value*, $\sigma^{2}=\sum_{d=1}^{D}\sigma_{d}^{2}$*and*$\omega^{2}=\sum_{d=1}^{D}\omega_{d}^{2}$.

##### Remark 1.

Under the similar assumptions, our convergence rate matches the rates of the distributed synchronize SGD and the distributed SGD with gradient compression and error-feedback [42]. Thus, we can further reduce computation and uplink communication from a full-length gradient update and communication [4, 42] to a single randomized block of the partial gradient update and communication without slowing down the convergence rate.

##### Nonsmooth regularization case.

This case corresponds to the execution of Line 9 in Algorithm 1. An appropriate optimally condition is based on the generalized gradient measure [10, 29, 30, 38]: ${\tilde{\text{G}}}_{\left(d\right)}[t]=\frac{1}{Y[t]}\left({\text{A}}_{\left(d\right)}-{\text{Prox}}_{Y[t],r_{d}}\left(A_{\left(d\right)}[t]-\gamma [t]\nabla_{{\text{A}}_{\left(d\right)}}F(\text{A}[t])\right)\right)$. The following theorem shows the convergence of Algorithm 1 for the nonsmooth regularization case.

###### Theorem 4.2.

*Suppose that Assumptions 4.1-4.6 hold. Let*$\left({\text{A}}_{\left(1\right)}[t],\ldots ,{\text{A}}_{\left(D\right)}[t]\right)$*be the iterates of Algorithm 1 with proximal operator (Line 9). Assume*$\gamma [t]=\frac{1}{4L}$. *We have*

(5) $$E\left[\sum_{d=1}^{D}\frac{1}{D}∥{\tilde{\text{G}}}_{\left(d\right)}[\text{Output}]{∥}_{F}^{2}\right]\leq \frac{16L}{T+1}\left(\text{Φ}(\text{A}[0])-{\text{Φ}}^{*}\right)\;\;+\frac{4\sigma^{2}}{DK}+\frac{32(1-\delta )}{D\delta^{2}}\left(\sigma^{2}+\omega^{2}\right),$$

*where*A[Output]*is sampled from*A[0]*to*A[T]*with uniform distribution*, $\text{Φ}(\text{A}[0])=F(\text{A}[0])+\sum_{d=1}^{D}r_{d}(\text{A}[0])$*and*${\text{Φ}}^{*}$*is the optimal value*.

##### Remark 2.

In the nonsmooth regularization case, the above convergence result is weaker than the previous smooth case in that we only ensure the difference between the initial loss and the optimal value will get smaller, but the generalized gradient is not guaranteed to approach 0 given that the variance and gradient norm related terms will dominate with increasing *T*. However, our empirical results show that the algorithm is able to converge to small losses.

#### 4.1.2 Convergence Analysis of Algorithm 2.

##### 

Now, we provide the convergence rate of Algorithm 2 by extending the proof in [4] to the block randomized setting, which is obtained under the same assumptions with Theorem 4.1. The main idea for the analysis is to introduce the virtual sequence of ${\tilde{\text{A}}}_{\left(d\right)}^{avg}[t+1]={\tilde{\text{A}}}_{\left(d\right)}^{avg}[t]-\gamma [t]\frac{1}{K}\sum_{k=1}^{R}G_{\left(d\right)}^{k}[t]$ and build an iterative descent relation for it. Meanwhile, we keep track of the error between the true and virtual averages of ${\text{A}}_{\left(d\right)}^{avg}[t]-{\tilde{\text{A}}}_{\left(d\right)}^{avg}[t]$, and the deviation between the local variables and the true average of ${\text{A}}^{avg}[t]-{\text{A}}^{k}[t]$. Since both deviations are well-bounded, it means ${\text{A}}^{k}[t],{\text{A}}^{avg}[t],{\tilde{\text{A}}}_{\left(d\right)}^{avg}[t]$ are close to each other. Finally, we can obtain the convergence result for the true sequence ${\text{A}}^{k}[t]$ by substituting the deviations into the descent relation obtained for ${\tilde{\text{A}}}_{\left(d\right)}^{avg}[t]$.

###### Theorem 4.3.

*Suppose that Assumptions 4.1-4.5 hold. Let*$\left({\text{A}}_{\left(1\right)}^{k}[t],\ldots ,{\text{A}}_{\left(D\right)}^{k}[t]\right)$*be the iterates of Algorithm 2, for k* = 1,...,*K and t* = 0,...,*T. Let* $\gamma [t]=\frac{C}{\sqrt{T+1}}$ *with* $0<C\leq \frac{1}{L}$. *We have*

$$\begin{array}{l} E\left[\sum_{d=1}^{D}\frac{1}{D}∥\nabla_{{\text{A}}_{\left(d\right)}}F(\text{A}[\text{Output}]){∥}_{F}^{2}\right]\leq \left(4C\left[F(\text{A}[0])-F^{*}\right]+2CL\sigma^{2}\right)\frac{1}{\sqrt{T+1}}\\ +\left(\frac{32C^{2}L^{2}\left(1-\delta^{2}\right)\left(\sigma^{2}+\omega^{2}\right)}{D\delta^{2}}+\frac{8C^{2}L^{2}\left(\sigma^{2}+\omega^{2}\right)}{DK}\right)\frac{\tau^{2}}{T+1}, \end{array}$$

*where* $\text{A[Output}]=\left({\text{A}}_{\left(1\right)}[\text{Output}],\ldots ,{\text{A}}_{\left(D\right)}[\text{Output}]\right)$ *is sampled from* ${\text{A}}^{k}[0]$ *to* ${\text{A}}^{k}[T]$, *for all k* = 1,...,*K, with uniform distribution*, $F^{*}$ *is the optimal value*, $\sigma^{2}=\sum_{d=1}^{D}\sigma_{d}^{2}$ *and* $\omega^{2}=\sum_{d=1}^{D}\omega_{d}^{2}$.

##### Remark 3.

Algorithm 2 maintains the same convergence rate of $O\left(\frac{1}{\sqrt{T+1}}\right)$ as Algorithm 1, despite the periodic communication. The communication gap *τ* only affects the term with order $O\left(\frac{1}{\sqrt{T+1}}\right)$, which is insignificant compared to the $O\left(\frac{1}{\sqrt{T+1}}\right)$ overall convergence rate. Thus, without increasing the iteration complexity, the periodic communication can further reduce communication cost.

### 4.2 Complexity Analysis

We provide the computation, storage and communication complexities for FedGTF-EF and FedGTF-EF-PC given |S| fibers being sampled by each client and the rank of the GTF being *R*.

#### Computational Complexity.

Our method is very efficient when compared to the following methods: 1) the classic CP-ALS and the full gradient descent-based GTF, which cost $O\left(DR\prod_{d=1}^{D}I_{d}\right)$; 2) the sampled randomized CP-ALS in [5] and SGD-based GTF in [15] with the same number of elements sampled, which cost $O\left(RS|\sum_{d=1}^{D}I_{d}\right)$; and 3) the same complexity as the full precision block randomized SGD-based TF [10].

##### Theorem 4.4.

*The per-iteration computational complexity of Algorithm****FedGTF-EF****and****FedGTF-EF-PC****for each client is*$O\left(\frac{1}{D}\left(\sum_{d=1}^{D}I_{d}\right)R|S|\right)$.

#### Communication Complexity.

Assume we are using the Sign compressor and comparing with full precision distributed SGD with all blocks communicated. Let *D* = 4, *τ* = 8, **FedGTF-EF** and **FedGTF-EF-PC** reduces up to 99.22% and 99.90% uplink communications. In general, we have:

##### Theorem 4.5.

***FedGTF-EF*** *reduces up to* $1-\frac{1}{32D}$ *uplink communication and* ***FedGTF-EF-PC*** *reduces up to* $1-\frac{1}{32D\tau }$ *uplink communication.*

#### Storage Complexity.

The fiber sampling based stochastic partial gradient avoids forming the whole element-wise partial gradient tensor Y, which reduces the storage for this step from $O\left(\prod_{d=1}^{D}I_{d}\right)$ to $O\left(|S|\frac{1}{D}\sum_{d=1}^{D}I_{d}\right)$, thus achieving the same cost efficiency with sampling-based random CP-ALS [5], full precision SGD [15] and block randomized full precision SGD [10].

## 5 EXPERIMENT

### 5.1 Experimental Setup

#### Datasets.

We consider two real-world EHR datasets^1^, as well as a synthetic dataset, which are introduced below,

- **CMS [1]** : A publicly available healthcare dataset with patients’ information protected. We adopt the rules in [20] to select the top 500 frequently observed diagnoses, procedures, and medications to form a 4th order tensor of size 125, 961 × 500 × 500 × 500 and a 3rd order tensor of size 91999 × 500 × 500 (with medication mode omitted).
- **MIMIC-III [16]** : It is a publicly available relational dataset that describes the patients information of the Intensive Care Units (ICUs). Similar to CMS dataset, we form a 4 mode tensor representing patients-diagnoses-procedures-medications with size 34, 272×500×500 × 500.
- **Synthetic data :** Synthetic data with size 4000×500×500×500 is generated as follows: for the nonzero entries, their values are sampled from uniform distribution for the least square loss setting and from binomial distribution for the logit loss setting, while positions of the non-zero entries are the same for both loss settings which are uniformly sampled from all entries with 10^−4^ non-zero ratio.

#### Algorithms for comparison.

We consider two different loss functions: the Bernoulli logit loss $f_{logit}$ and the least square loss. For the Bernoulli logit loss, we compare with: i) **GCP** (centralized) [22]; ii) **BrasCPD** (centralized) [10]; iii) **Centralized versions of FedGTF-EF**, iv) **FedGTF-EF-cyclic** and v) **FedGTF-EF-prox**. For the least square loss, we compare with: i) **BrasCPD** (centralized) [10]; ii) **FlexiFact** [6, 12]: a distributed tensor factorization algorithm; iii) **TRIP** [20]: a federated tensor factorization algorithm optimized with ADMM, which has deterministic per-iteration update solved in closed-form; iv) **DPFact** [27]: a federated SGD algorithm designed for collaborative tensor factorization. For fair comparison, we remove the differential privacy part of DPFact and substitute the *l*_2,1_ regularization with the *l*_1_ regularization as a new variant, DPFact-prox.

#### Ablation study.

We conduct ablation studies to illustrate the contribution of each communication reduction mechanism to the overall communication efficiency, which includes i) DistBrasCPD: the distributed version of BrasCPD [10] or FGTF with only the block-randomized technique; ii) DistBrasCPD-comp: FGTF with both block-randomized and gradient compression techniques; iii) DistSGDEF: distributed SGD with error-feedback that communicates full gradients and all blocks; iv) DistSGD-EF-comp: DistSGD-EF with gradient compression. Table 2 summarize the comparison with the proposed algorithms.

For our proposed algorithms, in addition to FedGTF-EF and FedGTF-EF-PC, we consider two variants: FedGTF-EF-cyclic (a variant of FedGTF-EF with cyclic mode updates), FedGTF-EF-prox (FedGTF-EF with *l*_1_ regularization). We vary the value of *τ* in {2, 4, 6, 8} for FedGTF-EF-PC.

#### Experiment results.

**Figure 4:**
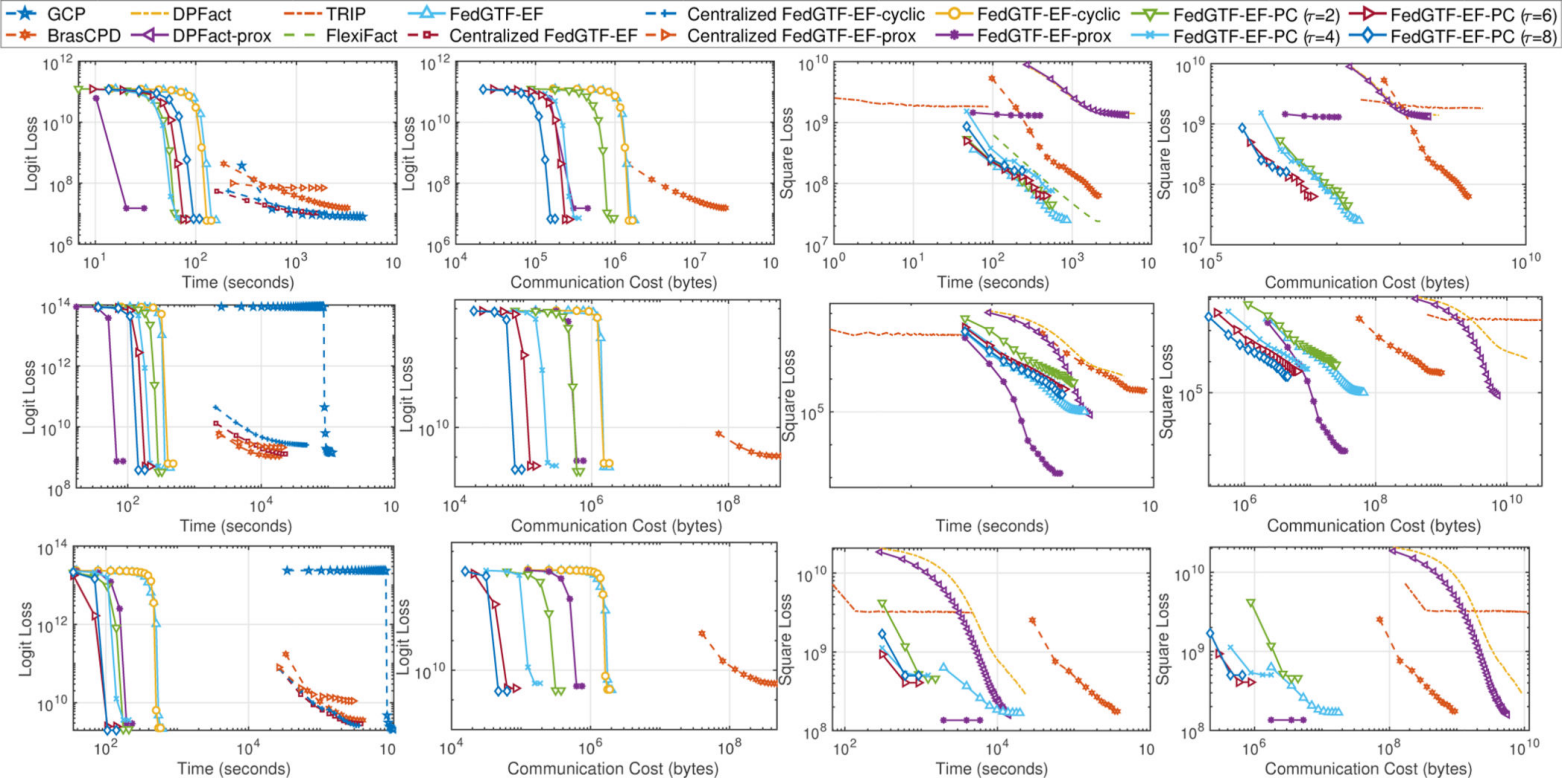
Loss decrease with respect to 1) computation time measured by seconds (column 1, 3 for Bernoulli Logit Loss and Least Square Loss respectively); 2) uplink communication cost measured by number of bytes (column 2, 4 for Bernoulli Logit Loss and Least Square Loss respectively). Top: 3-rd order CMS; Middle: 4-th order CMS; Bottom: MIMIC-III.

Our experiments show that FedGTF-EF and FedGTF-EF-PC are able to greatly improve the communication efficiency without slowing down the convergence and deteriorating the factorization quality. In detail, we have the following four observations: **i)** FedGTF-EF and its variants reduce loss faster with much less communication cost, for both the Bernoulli Logit Loss (Fig. 4 first two columns) and the Least Square loss (Fig. 4 last two columns) compared to the baseline methods. The communication cost per communication round is further reduced by increasing the local update iterations *τ* from 2 to 8 without hurting the performance of the Bernoulli logit loss and with a slightly worse loss for the least square loss. **ii)** FedGTF-EF, FedGTF-EF-PC and their variants are computationally efficient due to the fiber-sampling technique, i.e., they use lower computation cost compared to the baselines. By Fig. 4, for both objective functions, FedGTF-EF-PC, FedGTF-EF and its variants converges to similar losses as their centralized counterparts, while cost less time because more workers are involved in the updating process for the federated setting. Note that although TRIP converges faster in terms of time, but it tends to be trapped into bad local minima caused potentially by its deterministic per-iteration update. **iii)** FedGTF-EF, FedGTF-EF-PC and their variants converge to similar losses as the centralized counterpart, which indicates communication efficiency can be improved without sacrificing the factorization quality. **iv)** FedGTF-EF and FedGTF-EF-PC converge faster in terms of running time with more workers. As shown in Fig. 4 upper left and Fig. 6, with the number of workers increased from 8 to 16, the time for FedGTF-EF to converge reduces 65.58%.

**Figure 5:**
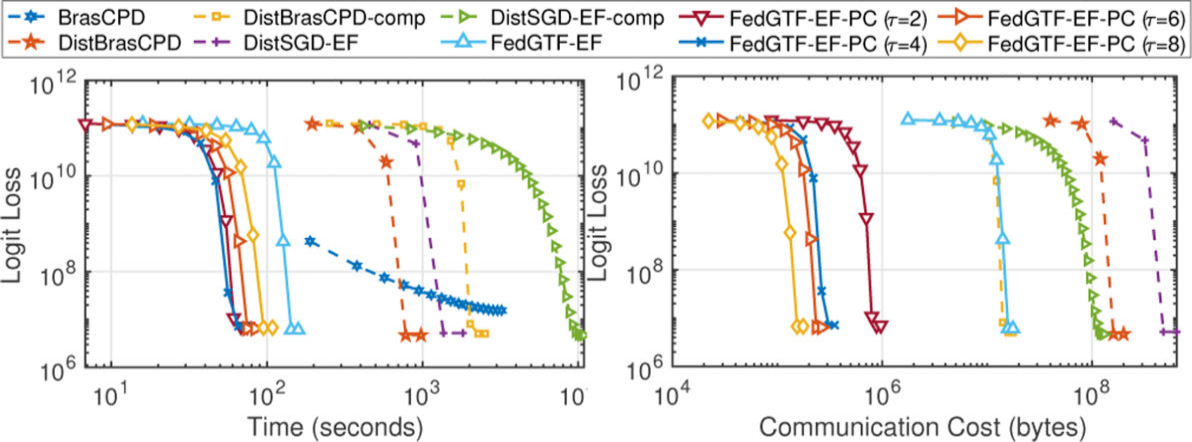
Ablation Study on 3-rd order CMS for Bernoulli Logit Loss.

**Figure 6:**
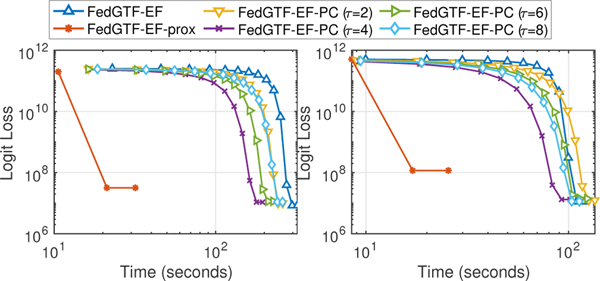
Comparison of different number of workers on 3rd order CMS for Bernoulli Logit Loss.

From the ablation study (Fig. 5), we can see: **i)** Block-randomized update and gradient compression can greatly reduce the communication cost by 75.00% and 96.88%, respectively. Therefore, gradient compression plays a more important role in communication reduction. **ii)** With both block-randomized and gradient compression, FedGTF-EF achieves a gradient reduction of 98.90% over FGTF. **iii)** Periodic communication further reduces the communication cost over FGTF by 99.94%, 99.97, 99.98%, and 99.99% with {2, 4, 6, 8} rounds of local communications respectively.

**Figure 7:**
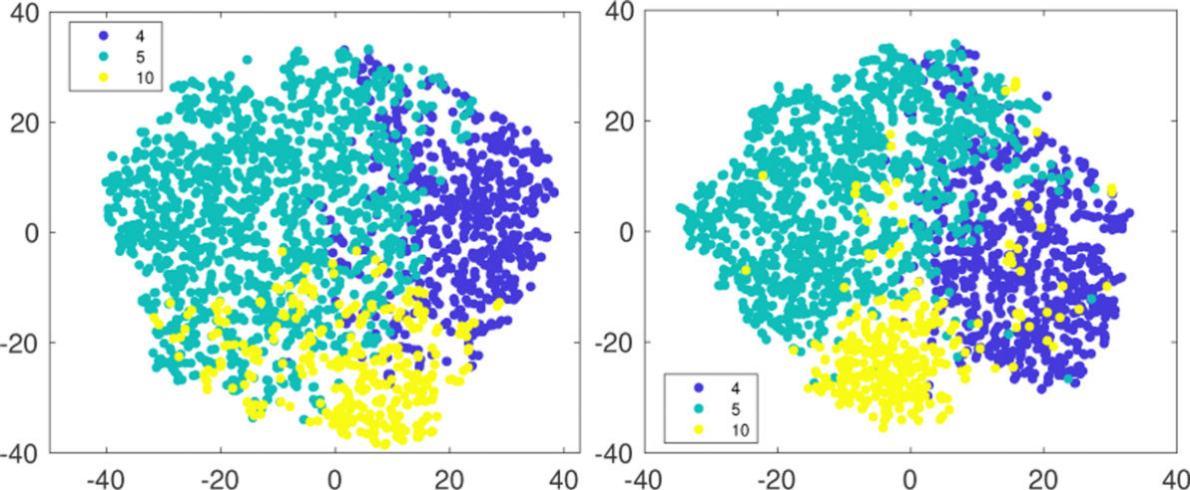
tSNE visualization of the patient representation learned by BrasCPD (left) and FedGTF-EF-PC(*τ* = 8) (right). Each point represents a patient which is colored according to the highest-valued coordinate in the patient representation vector among the top 3 phenotypes extracted based on the factor weights $\lambda_{r}={\left\|{\text{A}}_{\left(1\right)}(:,r)\right\|}_{F}{\left\|{\text{A}}_{\left(2\right)}(:,r)\right\|}_{F}\cdots {\left\|{\text{A}}_{\left(D\right)}(:,r)\right\|}_{F}$.

Finally, we evaluate the quality of the federated factorization factors by considering the patient subgroup identification following [28], as illustrated in Fig. 7. We use tSNE to map the *R* dimensional vectors into the 2 dimensional space. We first identify the top 3 phenotypes that have the largest factor weights, which are the phenotypes #4, #5, #10 in Fig. 7 (phenotype details are shown in Table 3). Then, we color the patients by assigning each patient to one of the top 3 phenotypes using the largest patient weight among the top 3 along the representation vector. Fig. 7 shows FedGT-FEF-PC with *τ* = 8 local updates has comparable performance to the centralized baseline BrasCPD in clustering the patients with the same phenotype together. This demonstrates that our method can achieve communication compression without sacrificing the factorization quality.

## 6 CONCLUSION

In this paper, we study the under explored communication efficiency problem in federated (more broadly the distributed) generalized tensor factorization for collaborative phenotyping. We propose FedGTF-EF with communication efficient designs of block randomized update and gradient compression with error-feedback, which encompassed two levels of uplink communication reduction: reduced number of blocks and reduced per-element communication. We further reduce the communication rounds by periodic averaging to develop the FedGTF-EF-PC algorithm. The convergence guarantee is provided under common assumptions applied not only to generalized tensor factorization problems but also to more general machine learning problems possessing a multi-block structure. Our algorithm can maintain low computational and storage complexity while occupying much lower uplink communication cost. We demonstrate its superior efficiency and uncompromised quality on synthetic and two real-world EHR datasets.

## Figures and Tables

**Table 1:** Symbols and notations used in this paper

Symbol	Definition
x, X, X	Vector, Matrix, Tensor
$X_{<d>}$	Mode-*d* matricization of X
$∥\cdot {∥}_{1}$	*ℓ*_1_-norm
$∥\cdot {∥}_{F}$	Frobenius norm
⊛	Hadamard (element-wise) multiplication
⊙	Khatri Rao product
∘	Outer product
〈⋅,⋅〉	Inner product

**Table 2:** Comparison of algorithms in ablation study.

Algorithm	Element-level Reduction	Block-level Reduction	Round-level Reduction	Convergence Guarantee	Compression Ratio
DistBrasCPD	✗	✓	✗	✗	1 − 1/*D*
DistBrasCPD-comp	✓	✓	✗	✗	1 − 1/32*D*
DistSGD-EF	✗	✗	✗	✗	0
DistSGD-EF-comp	✓	✗	✗	✗	1 − 1/32
FedGTF-EF	✓	✓	✗	✓	1 − 1/32*D*
FedGTF-EF-PC	✓	✓	✓	✓	1 − 1/32*Dτ*

**Table 3:** Top 3 phenptypes extracted by FedGTF-EF-PC(*τ* = 8) on MIMIC-III data. Red, blue, and green indicate diagnoses, procedures, and medication, respectively.

P10: Diabetic Heart Failure
Diabetes mellitus without mention of complication
Background diabetic retinopathy
Acute systolic heart failure
Acute on chronic systolic heart failure
Chronic diastolic heart failure
Acute on chronic combined systolic and diastolic heart failure
Insertion of one vascular stent
Open heart valvuloplasty of tricuspid valve without replacement
Operations on other structures adjacent to valves of heart
(Aorto)coronary bypass of three coronary arteries
Captopril (ACE inhibitor), Insulin, Pyridostigmine Bromide, Isosorbide Dinitrate
P5: Hypertensive Heart Failure

Pure hypercholesterolemia
Cardiac tamponade
Ventricular fibrillation
Cardiac arrest
Acute systolic heart failure
Percutaneous insertion of carotid artery stent(s)
Pericardiocentesis
Extracorporeal circulation auxiliary to open heart surgery
Other endovascular procedures on other vessels
Rosuvastatin Calcium, Isosorbide Dinitrate, Hydrochlorothiazide, Digoxin, Clonidine HCl
P4: Peripheral Arterial Disease

Congestive heart failure
Atherosclerosis of native arteries of the extremities
– with intermittent claudication
Acute venous embolism and thrombosis of
–superficial veins of upper extremity
Insertion of drug-eluting coronary artery stent(s)
(Aorto)coronary bypass of two coronary arteries
Interruption of the vena cava
Suture of artery
Angioplasty of other non-coronary vessel(s)
Carvedilol, Metoprolol succinate, Amiodarone HCl, Nitroglycerin, Calcium Chloride

**Figure 8:**
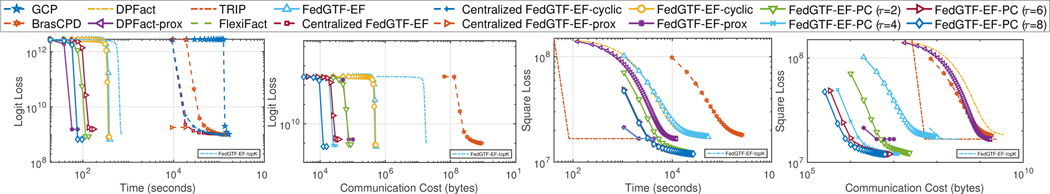
Bernoulli Logit Loss and Square Loss with respect to computation time and communication for synthetic data.

**Figure 9:**
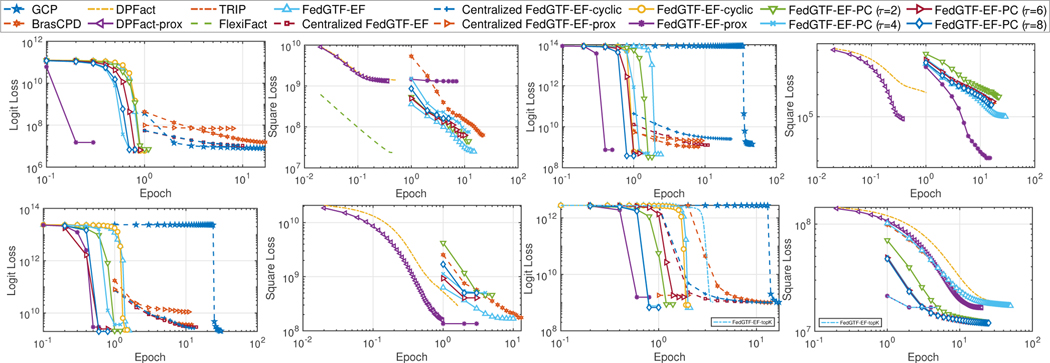
Bernoulli logit loss (column 1,2) and Least Square loss (column 3,4) decrease with respect to epochs.

## CCS CONCEPTS

• Information systems → Data extraction and integration; • Applied computing → Health informatics;

## Footnotes

1 Code available at: https://github.com/jma78/FedGTF-EF

## APPENDIX

### A ADDITIONAL MATERIALS FOR EXPERIMENTS

#### A.1 Parameter Settings

For MIMIC-III, CMS and synthetic datasets, each algorithm is run for 500 iterations per epoch until converge, while for delicious dataset, each algorithm is run for 1000 iterations per epoch. For GCP algorithm, we tune the stepsize within the range of {10^−8^, 10^−9^, 10^−10^, 10^−11^}, while for the rest algorithms, we tune the stepsize by grid search through {2^2^, 2^1^, 2^0^, 2^−1^, 2^−2^,..., 2^−11^}. The parameter for the proximal operator is set to 10^−4^ for all the algorithms with the proximal operators (FedGTF-EF-prox, DPFact-prox). For all the federated algorithms, we by default horizontally partition the tensor (along *I*_1_ mode) into 8 tensors without overlapping and distribute each of them to 8 client nodes respectively. We also test different numbers of workers (16 workers and 32 workers), where the stepsizes are set to the same as for 8 workers. The best stepsizes for each algorithms for different datasets are set as in Table 4 and 5.

Each experiment is averaged over 5 repetitions. All experiments are run on Matlab 2019a on an r5.12xlarge instance of AWS EC2 with Tensor Toolbox Version 3.1 [3].

#### A.2 Additional Experiments

Two additional groups of figures are presented here. Fig. 8 shows the loss decrease for both the Bernoulli loss and the Least Square loss with respect to time and communication for the synthetic data. Fig. 9 shows the Bernoulli loss and the Least Square loss decrease with respect to epochs in supplementary to the figures showed in the main paper with respects to time and communication. Similar conclusions can be drawn with the real-world EHR datasets in the main paper. That is, the proposed algorithms achieve more efficient convergence than the centralized baselines under the Bernoulli logit loss and the distributed baseline under the least square loss. It is also more communication-efficient than the algorithms without gradient compressor (BrasCPD distributed version) and without the block randomized mechanism (DPFact and its variants).

**Table 4:** Best Stepsizes for the Bernoulli Logit Loss

Algorithm	MIMIC-III	4th order CMS	3rd order CMS	Synthetic

GCP	10^−10^	10^−10^	10^−10^	10^−9^
BrasCPD	2^−4^	2^−1^	2^−4^	2^−5^
Centralized FedGTF-EF	2^−3^	2^−1^	2^−2^	2^−4^
Centralized FedGTF-EF-cyclic	2^−2^	2^−2^	2^−2^	2^−4^
Centralized FedGTF-EF-prox	2^−2^	2^−0^	2^−2^	2^−2^
FedGTF-EF	2^−3^	2^−2^	2^−2^	2^−4^
FedGTF-EF-cyclic	2^−4^	2^−2^	2^−2^	2^−4^
FedGTF-EF-prox	2^−2^	2^−3^	2^−4^	2^−1^
FedGTF-EF-PC(*τ* = 2)	2^−5^	2^−5^	2^−2^	2^−4^
FedGTF-EF-PC(*τ* = 4)	2^−5^	2^−5^	2^−2^	2^−4^
FedGTF-EF-PC(*τ* = 6)	2^−5^	2^−5^	2^−2^	2^−4^
FedGTF-EF-PC(*τ* = 8)	2^−5^	2^−5^	2^−2^	2^−4^

**Table 5:** Best Stepsizes for the Least Square Loss

Algorithm	MIMIC-III	4-th order CMS	3-rd order CMS	Synthetic

BrasCPD	2^−5^	2^0^	10^−4^	2^−2^
FlexiFact	-	-	2	-
DPFact	2^−4^	2^1^	2^−10^	2^−2^
DPFact-prox	2^−4^	2^1^	2^−10^	2^−2^
FedGTF-EF	2^−4^	2^0^	2^−11^	2^−2^
FedGTF-EF-prox	2^−5^	2^0^	2^−10^	2^−2^
FedGTF-EF-PC(*τ* = 2)	2^−4^	2^0^	2^−10^	2^−2^
FedGTF-EF-PC(*τ* = 4)	2^−4^	2^0^	2^−10^	2^−2^
FedGTF-EF-PC(*τ* = 6)	2^−4^	2^0^	2^−1^0	2^−2^
FedGTF-EF-PC(*τ* = 8)	2^−4^	2^0^	2^−10^	2^−2^

### B CONVERGENCE ANALYSIS OF ALGORITHM 1

#### B.1 Proof Sketch of Theorem 4.1

##### B.1.1 Auxiliary variables for the proof and iterative relation.

The following auxillary variables and virtual iterations are introduced only for the proof: ${\tilde{\text{A}}}_{\left(d\right)}[t]:={\text{A}}_{\left(d\right)}[t]-\frac{1}{K}\sum_{k=1}^{K}{\text{E}}_{\left(d\right)}^{k}[t]$. Given the auxiliary variable ${\tilde{\text{A}}}_{\left(d\right)}[t]$, we have the following iterative relation: if $d=d_{\xi [t]},{\tilde{\text{A}}}_{\left(d\right)}[t+1]={\tilde{\text{A}}}_{\left(d\right)}[t]-\gamma [t]\frac{1}{K}\sum_{k=1}^{K}{\text{G}}_{\left(d\right)}^{k}[t]$; else if $d\neq d_{\xi [t]},{\tilde{\text{A}}}_{\left(d\right)}[t+1]={\tilde{\text{A}}}_{\left(d\right)}[t]$.

##### B.1.2 Additional Lemma.

The following lemma extends Lemma 3 in [19] to our block randomized case.

###### Lemma B.1.

*(Bounding the expectation of the block-wise feedback error averaged among client nodes) For d* = 1,...,*D and for t* = 0,...,*T, assuming constant step size* γ[t]=γ, *we have*

(6) $$E\left[{\left\|\frac{1}{K}\sum_{k=1}^{K}{\text{E}}_{\left(d\right)}^{k}[t+1]\right\|}_{F}^{2}\right]\leq \frac{4(1-\delta )}{\delta^{2}}\gamma^{2}\left(\sigma_{d}^{2}+\omega_{d}^{2}\right).$$

##### B.1.3 Main proof sketch of Theorem 4.1.

By block-wise Lipschitz smoothness assumption of the loss function:

$$\begin{array}{c} F(\tilde{\text{A}}[t+1])\leq F(\tilde{\text{A}}[t])-\gamma [t]\langle \nabla_{{\text{A}}_{\left(d_{\xi }[t]\right)}}F(\tilde{\text{A}}[t]),\frac{1}{K}\sum_{k=1}^{K}{\text{G}}_{\left(d_{\xi }[t]\right)}^{k}[t]\rangle \\ +\frac{L_{d_{\xi }[t]}{\left(\gamma [t]\right)}^{2}}{2}{\left\|\frac{1}{K}\sum_{k=1}^{K}{\text{G}}_{\left(d_{\xi }[t]\right)}^{k}[t]\right\|}_{F}^{2}. \end{array}$$

By Assumption 4.2 that $E_{\zeta [t]}\left[\frac{1}{K}\sum_{k=1}^{K}{\text{G}}_{d_{\xi [t]}}^{k}[t]|F[t],\xi [t]\right]=\nabla_{{\text{A}}_{(d_{\xi [t]})}}F(\text{A}[t])$, we have

(7) $$\begin{array}{l} {\text{E}}_{\zeta [t]}\left[{\left\|\frac{1}{K}\sum_{k=1}^{K}{\text{G}}_{\left(d_{\xi }[t]\right)}^{k}[t]-\nabla_{{\text{A}}_{\left(d_{\xi [t]}\right)}}F(\text{A}[t])\right\|}_{F}^{2}|F[t],\xi [t]\right]\\ =E_{\zeta [t]}\left[{\left\|\frac{1}{K}\sum_{k=1}^{K}{\text{G}}_{\left(d_{\xi }[t]\right)}^{k}[t]\right\|}_{F}^{2}|F[t],\xi [t]\right]-\nabla {\left\|{}_{{\text{A}}_{\left(d_{\xi }[t]\right)}}F(\text{A}[t])\right\|}_{F}^{2}. \end{array}$$

Taking conditional expectation on both sides of eq.(B.1.3) with respect to filtration F[t] and randomness of ζ[t] during the stochastic gradient computation and plugging eq.(7) in, we have

$$\begin{array}{l} E_{\zeta [t]}[F(\tilde{\text{A}}[t+1])|F[t],\xi [t]]\\ \leq F(\tilde{\text{A}}[t])-\gamma [t]\left(1-\frac{L_{d_{\xi }[t]}\gamma [t]}{2}\right){\left\|\nabla_{{\text{A}}_{\left(d_{\xi [t]}\right)}}F(\text{A}[t])\right\|}_{F}^{2}\\ +\frac{L_{d_{\xi }[t]}{\left(\gamma [t]\right)}^{2}}{2K}\sigma_{d_{\xi }[t]}^{2}\\ +\gamma [t]\langle \nabla_{{\text{A}}_{\left(d_{\xi [t]}\right)}}F(\text{A}[t])-\nabla_{{\text{A}}_{\left(d_{\xi }[t]\right)}}F(\tilde{\text{A}}[t]),\nabla_{{\text{A}}_{(d_{\xi [t]})}}F(\text{A}[t])\rangle . \end{array}$$

We bound $\langle \nabla_{{\text{A}}_{(d_{\xi [t]})}}F(\text{A}[t])-\nabla_{{\text{A}}_{\left(d_{\xi }[t]\right)}}F(\tilde{\text{A}}[t]),\nabla_{{\text{A}}_{(d_{\xi [t]})}}F(\text{A}[t])\rangle$ by Young’s inequality, we have

$$\begin{array}{l} E_{\zeta [t]}[F(\tilde{\text{A}}[t+1])|F[t],\xi [t]]\\ \leq F(\tilde{\text{A}}[t])-\gamma [t]\left(1-\frac{L_{d_{\xi }[t]}\gamma [t]+\rho }{2}\right){\left\|\nabla_{{\text{A}}_{\left(d_{\xi }[t]\right)}}F(\text{A}[t])\right\|}_{F}^{2}\\ +\frac{L_{d_{\xi }[t]}{\left(\gamma [t]\right)}^{2}}{2K}\sigma_{d_{\xi }[t]}^{2}+\frac{L_{d_{\xi }[t]}^{2}\gamma [t]}{2\rho }{\left\|\frac{1}{K}\sum_{k=1}^{K}{\text{E}}_{\left(d_{\xi [t]}\right)}^{k}[t]\right\|}_{F}^{2}. \end{array}$$

Taking expectation with respect to *ξ*[*t*] conditioned on F[t] and substituting $L=\max\left\{L_{1},\ldots ,L_{D}\right\},\sigma^{2}=\sum_{d=1}^{D}\sigma_{d}^{2}$ in, we have

$$\begin{array}{l} E_{\xi [t]}[F(\tilde{\text{A}}[t+1])|F[t]]\\ \leq F(\tilde{\text{A}}[t])-\gamma [t]\left(1-\frac{L_{\gamma }[t]+\rho }{2}\right)\frac{1}{D}\sum_{d=1}^{D}{\left\|\nabla_{{\text{A}}_{\left(d\right)}}F(\text{A}[t])\right\|}_{F}^{2}\\ +\frac{L{\left(\gamma [t]\sigma \right)}^{2}}{2KD}+\frac{L^{2}\gamma [t]}{2\rho }\frac{1}{D}\sum_{d=1}^{D}{\left\|\frac{1}{K}\sum_{k=1}^{K}{\text{E}}_{\left(d\right)}^{k}[t]\right\|}_{F}^{2}. \end{array}$$

By Lemma B.1 and let *γ*[*t*] = *t*, we have

(8) $$\begin{array}{l} E_{\xi [t]}[F(\tilde{\text{A}}[t+1])|F[t]]\\ \leq F(\tilde{\text{A}}[t])-\gamma \left(1-\frac{L\gamma +\rho }{2}\right)\frac{1}{D}\sum_{d=1}^{D}{\left\|\nabla_{{\text{A}}_{\left(d\right)}}F(\text{A}[t])\right\|}_{F}^{2}\\ +\frac{L{\left(\gamma \sigma \right)}^{2}}{2KD}+\frac{2L^{2}\gamma^{3}(1-\delta )\left(\sigma^{2}+\omega^{2}\right)}{\rho D\delta^{2}}. \end{array}$$

Taking total expectation with respect to all the random variables in F[t], and averaging the above from *t* = 0 to *T* and letting *ρ* < 2−*Lγ*, $F^{*}$ the optimal value, we have we have

(9) $$\begin{array}{l} \frac{1}{T+1}\sum_{T}^{t=0}E\left[\frac{1}{D}\sum_{d=1}^{D}{\left\|\nabla_{{\text{A}}_{\left(d\right)}}F(\text{A}[t])\right\|}_{F}^{2}\right]\\ \leq \frac{1}{(T+1)\gamma \left(1-\frac{L\gamma +\rho }{2}\right)}\left[F(\text{A}[0])-F^{*}\right]\\ +\frac{1}{\left(1-\frac{L\gamma +\rho }{2}\right)}\left[\frac{L\gamma \sigma^{2}}{2KD}+\frac{2L^{2}\gamma^{2}(1-\delta )\left(\sigma^{2}+\omega^{2}\right)}{\rho D\delta^{2}}\right]. \end{array}$$

By setting *ρ* = 1 and using $E\left[\frac{1}{D}\sum_{d=1}^{D}∥\nabla_{{\text{A}}_{\left(d\right)}}F(\text{A}[\text{Output}]){∥}_{F}^{2}\right]\leq \sum_{t=0}^{T}\frac{1}{T+1}E\left[\frac{1}{D}\sum_{d=1}^{D}{\left\|\nabla_{{\text{A}}_{\left(d\right)}}F(\text{A}[t])\right\|}_{F}^{2}\right]$, letting $\gamma =\min\left\{\frac{1}{2L},\frac{\varrho }{\sqrt{T+1}/\sqrt{K}+\frac{{\left(1-\delta \right)}^{1/3}}{\delta^{2/3}}T^{1/3}}\right\}$ for some *ϱ* > 0, we complete the proof of Theorem 4.1:

$$\begin{array}{l} E\left[\frac{1}{D}\sum_{d=1}^{D}∥\nabla_{{\text{A}}_{\left(d\right)}}F(\text{A}[\text{Output}]){∥}_{F}^{2}\right]\\ \leq \frac{8L}{T+1}\left(F(\text{A}[0])-F^{*}\right)+\left[\frac{4}{\varrho }\left(F(\text{A}[0])-F^{*}\right)+\frac{2L\sigma^{2}\varrho }{D}\right]\frac{1}{\sqrt{M(T+1)}}\\ +\left[\frac{4}{\varrho }\left(F(\text{A}[0])-F^{*}\right)+\frac{8L^{2}\varrho^{2}\left(\sigma^{2}+\omega^{2}\right)}{D}\right]\frac{{\left(1-\delta \right)}^{1/3}}{\delta^{2/3}{\left(T+1\right)}^{2/3}}. \end{array}$$

#### B.2 Proof Sketch of Theorem 4.2

##### B.2.1 Auxiliary variables for the proof and iterative relation.

We derive the convergence by regarding the iteration as using inexact gradient, which is different from the approach used for the smooth case which is regarded as using delayed variable:

$$\begin{array}{l} {\text{A}}_{\left(d_{\xi }[t]\right)}[t+1]=\text{Prox}\left({\text{A}}_{\left(d_{\xi }[t]\right)}[t]-\frac{1}{K}\sum_{k=1}^{K}{\text{Δ}}_{\left(d_{\xi }[t]\right)}^{k}[t]\right)=\\ \text{Prox}({\text{A}}_{\left(d^{k}\right)}[t]-\gamma [t]\frac{1}{K}\sum_{k=1}^{K}({\text{G}}_{\left(d_{\xi }[t]\right)}^{k}[t]\\ +\frac{1}{\gamma [t]}\left({\text{E}}_{\left(d_{\xi }[t]\right)}^{k}[t+1]-{\text{E}}_{\left(d_{\xi }[t]\right)}^{k}[t]\right))). \end{array}$$

We define the generalized gradient $\text{Z}[t]=({\text{Z}}_{\left(1\right)}[t],\ldots ,\left({\text{Z}}_{\left(D\right)}[t]\right)$, where ${\text{Z}}_{\left(d\right)}[t]=\frac{1}{\gamma [t]}\left({\text{A}}_{\left(d\right)}[t]-{\text{Prox}}_{r_{\left(d\right)}}\left({\text{A}}_{\left(d\right)}[t]-\gamma [t]\nabla_{{\text{A}}_{\left(d\right)}}F(\text{A}[t])\right)\right)$ if $d=d_{\xi }[t],{\bar{\text{A}}}_{\left(d\right)}[t+1]={\text{Prox}}_{r_{\left(d\right)}}\left({\text{A}}_{\left(d\right)}[t]-\gamma [t]\nabla_{{\text{A}}_{\left(d\right)}}F(\text{A}[t])\right)$, else if $d\neq d_{\xi }[t]{\bar{\text{A}}}_{\left(d\right)}[t+1]={\text{A}}_{\left(d\right)}[t]$.

let Φ(A[t])=F(A[t])+r(A[t]).

##### B.2.2 Additional Lemma.

We need Lemma 1 from [30].

###### Lemma B.2.

*Let*$\text{y}={\text{Prox}}_{\gamma r}(\text{x}-\gamma \text{g})$, *for some* g. *Then for* y, *the following inequality holds for any* z,

(10) $$r(\text{y})+\langle \text{y}-\text{z},\text{g}\rangle \leq r(\text{z})+\frac{1}{2\gamma }\left[∥\text{z}-\text{x}{∥}_{2}^{2}-∥\text{y}-\text{x}{∥}_{2}^{2}-∥\text{y}-\text{z}{∥}_{2}^{2}\right].$$

##### B.2.3 Main Proof sketch of Theorem 4.2.

By the block-wise smoothness of *F*, the convexity of $r_{\left(d\right)}(\cdot )$, and the optimality of $\bar{\text{A}}\left(d_{\xi }[t]\right)[t+1]$ for ${\text{Prox}}_{r_{\left(d\right)}}\left({\text{A}}_{\left(d\right)}[t]-\gamma \nabla_{{\text{A}}_{\left(d\right)}}F(\text{A}[t])\right)$, we have

(11) $$\text{Φ}(\bar{\text{A}}[t+1])\leq \text{Φ}(\text{A}[t])+\left(\frac{L_{(d_{\xi }[t])}}{2}-\frac{1}{\gamma [t]}\right){\left\|{\bar{\text{A}}}_{\left(d_{\xi }[t]\right)}[t+1]-{\text{A}}_{\left(d_{\xi }[t]\right)}[t]\right\|}_{F}^{2}.$$

By Lemma B.2, we have

$$\begin{array}{l} F\left({\text{A}}_{\left(d_{\xi }[t]\right)}[t+1],{\text{A}}_{\left(-d_{\xi }[t]\right)}[t]\right)+r_{\left(d_{\xi }[t]\right)}\left({\text{A}}_{\left(d_{\xi }[t]\right)}[t+1]\right)\\ \leq F\left({\bar{\text{A}}}_{\left(d_{\xi }[t]\right)}[t+1],{\text{A}}_{\left(-d_{\xi }[t]\right)}[t]\right)+r_{\left(d_{\xi }[t]\right)}\left({\bar{\text{A}}}_{\left(d_{\xi }[t]\right)}[t+1]\right)\\ +\langle {\text{A}}_{\left(d_{\xi }[t]\right)}[t+1]-{\bar{\text{A}}}_{\left(d_{\xi }[t]\right)}[t+1],\nabla_{\left(d_{\xi }[t]\right)}F\left({\text{A}}_{\left(d_{\xi }[t]\right)}[t]\right)\\ -\frac{1}{K}\sum_{k=1}^{K}\left({\text{G}}_{\left(d_{\xi }[t]\right)}^{k}[t]+\frac{1}{\gamma [t]}\left({\text{E}}_{\left(d_{\xi }[t]\right)}^{k}[t+1]-{\text{E}}_{\left(d_{\xi }^{k}[t]\right)}[t]\right)\right)\rangle \\ +\left(\frac{L_{(d_{\xi }[t])}}{2}-\frac{1}{2\gamma [t]}\right){\left\|{\text{A}}_{\left(d_{\xi }[t]\right)}[t+1]-{\text{A}}_{\left(d_{\xi }[t]\right)}[t]\right\|}_{F}^{2}\\ +\left(\frac{L_{(d_{\xi }[t])}}{2}+\frac{1}{2\gamma [t]}\right){\left\|{\bar{\text{A}}}_{\left(d_{\xi }[t]\right)}[t+1]-{\text{A}}_{\left(d_{\xi }[t]\right)}[t]\right\|}_{F}^{2}\\ -\frac{1}{2\gamma [t]}{\left\|{\bar{\text{A}}}_{\left(d_{\xi }[t]\right)}[t+1]-{\text{A}}_{\left(d_{\xi }[t]\right)}[t+1]\right\|}_{F}^{2}. \end{array}$$

By bounding the third row of the above equation, choosing *ρ*_1_ = 2*γ*[*t*] and *ρ*_2_ = 2, with eq.(11), and letting $\gamma [t]\leq \frac{1}{2L_{\left(d_{\xi }[t]\right)}}$, we have

$$\begin{array}{l} \text{Φ}(\text{A}[t+1])\leq \text{Φ}(\text{A}[t])+\left(L_{\left(d_{\xi }[t]\right)}-\frac{1}{2\gamma [t]}\right){\left\|{\bar{\text{A}}}_{\left(d_{\xi }[t]\right)}[t+1]-{\text{A}}_{\left(d_{\xi }[t]\right)}[t]\right\|}_{F}^{2}\\ +\gamma [t]\frac{1}{K}\sum_{k=1^{K}}{\left\|\nabla_{\left(d_{\xi }[t]\right)}F\left({\text{A}}_{\left(d_{\xi }[t]\right)}[t]\right)-{\text{G}}_{\left(d_{\xi }[t]\right)}^{k}[t]\right\|}_{F}^{2}\\ +\frac{1}{\gamma [t]}\frac{1}{K}\sum_{k=1}^{K}{\left\|{\text{E}}_{\left(d_{\xi }[t]\right)}^{k}[t+1]-{\text{E}}_{\left(d_{\xi }[t]\right)}^{k}[t]\right\|}_{F}^{2}. \end{array}$$

Taking conditional expectation with respect to *ξ*[*t*] conditioned on filtration F[t], by Lemma B.1 and letting *γ*[*t*] = *t*, we have

$$\begin{array}{l} \text{Φ}(\text{A}[t+1])\leq \text{Φ}(\text{A}[t])+\left(L-\frac{1}{2\gamma }\right)\frac{1}{D}\sum_{d=1}^{D}∥\;∥{\bar{\text{A}}}_{\left(d\right)}[t+1]-{\text{A}}_{\left(d\right)}[t]{∥}_{F}^{2}\\ +\frac{\gamma \sigma^{2}}{D}+\frac{1}{D}\frac{8(1-\delta )}{\delta^{2}}\gamma \left(\sigma^{2}+\omega^{2}\right). \end{array}$$

Taking total expectation (i.e. with respect to all random variables in F[t]), averaging from *t* = 0 to *T* and using $E\left[\sum_{d=1}^{D}\frac{1}{D}∥{\tilde{\text{G}}}_{\left(d\right)}[\text{Output}]{∥}_{F}^{2}\right]\leq \frac{1}{T+1}E\left[\sum_{d=1}^{D}\frac{1}{D}{\left\|{\tilde{\text{G}}}_{\left(d\right)}[t]\right\|}_{F}^{2}\right]=\frac{1}{T+1}E\left[\sum_{d=1}^{D}\frac{1}{D}{\left\|\left(\bar{\text{A}}(d)[t+1]-{\text{A}}_{\left(d\right)}[t]\right)/\gamma \right\|}_{F}^{2}\right]$, by setting $\gamma =\frac{1}{4L}$, we complete our proof:

$$\begin{array}{l} E\left[\sum_{d=1}^{D}\frac{1}{D}∥{\tilde{\text{G}}}_{\left(d\right)}[\text{Output}]{∥}_{F}^{2}\right]\\ \leq \frac{16L}{T+1}\left(\text{Φ}(\text{A}[0])-{\text{Φ}}^{*}\right)+\frac{4\sigma^{2}}{DK}+\frac{32(1-\delta )}{D\delta^{2}}\left(\sigma^{2}+\omega^{2}\right). \end{array}$$
